# Construction of High-Quality Rice Ribosome Footprint Library

**DOI:** 10.3389/fpls.2020.572237

**Published:** 2020-09-04

**Authors:** Xiaoyu Yang, Jie Cui, Bo Song, Yu Yu, Beixin Mo, Lin Liu

**Affiliations:** ^1^Guangdong Provincial Key Laboratory for Plant Epigenetics, College of Life Sciences and Oceanography, Longhua Bioindustry and Innovation Research Institute, Shenzhen University, Shenzhen, China; ^2^Shenzhen Branch, Guangdong Laboratory for Lingnan Modern Agriculture, Genome Analysis Laboratory of the Ministry of Agriculture, Agricultural Genomics Institute at Shenzhen, Chinese Academy of Agricultural Sciences, Shenzhen, China

**Keywords:** *Oryza sativa*, high-quality, high-throughput sequencing, ribosome footprint library, translation

## Abstract

High-throughput sequencing of ribosome footprints precisely maps and quantifies *in vivo* mRNA translation. The ribosome footprint sequencing has undergone continuing development since its original report. Here we provide a detailed protocol for construction of high-quality ribosome footprint library of rice. Rice total polysomes are isolated with a modified low ionic polysome extraction buffer. After nuclease digestion, rice ribosome footprints are extracted using SDS method followed by column purification. High-quality rice ribosome footprint library with peak reads of approximately 28-nucleotide (nt) length and strong 3-nt periodicity is constructed *via* key steps including rRNA depletion, end repair, 3’ adapter ligation, reverse transcription, circularization, PCR enrichment and several rounds of purification. Biological significance of rice ribosome footprint library is further revealed by the comparison of transcriptomic and translatomic responses to salt stress and the utilization for novel open reading frame (ORF) identification. This improved protocol for rice ribosome footprint library construction will facilitate the global comprehension and quantitative measurement of dynamic translation in rice.

## introduction

Translation of mRNAs, one of indispensable steps for gene expression, is directly associated with final proteome, which is profoundly involved in all aspects of cellular, physiological and developmental processes such as cell growth and division (Miettinen et al., 2019), organogenesis (Fujii et al., 2017), reproductivity (Sousa Martins et al., 2016), oncogenesis (Robichaud et al., 2019) and acclimation to variable environmental conditions (Merchante et al., 2017) in all kinds of organisms. To monitor gene expression, series of methodologies have been well developed from small-scale ones such as Northern blotting (Krumlauf, 1994), qRT-PCR (Nolan et al., 2006) and Western blotting (Burnette, 2011), to genome-wide ones such as microarray analysis (Slonim and Yanai, 2009), transcriptome (Zhang et al., 2018b) and protein sequencing (Timp and Timp, 2020). In contrast, the methodology that can directly evaluate mRNA translation is still greatly limited, though the crucial roles of translation regulation in gene expression have been well documented (Istomine et al., 2016; Merchante et al., 2017; Zhang et al., 2018c; Moissoglu et al., 2019; Park and Subramaniam, 2019).

**Figure 1:**
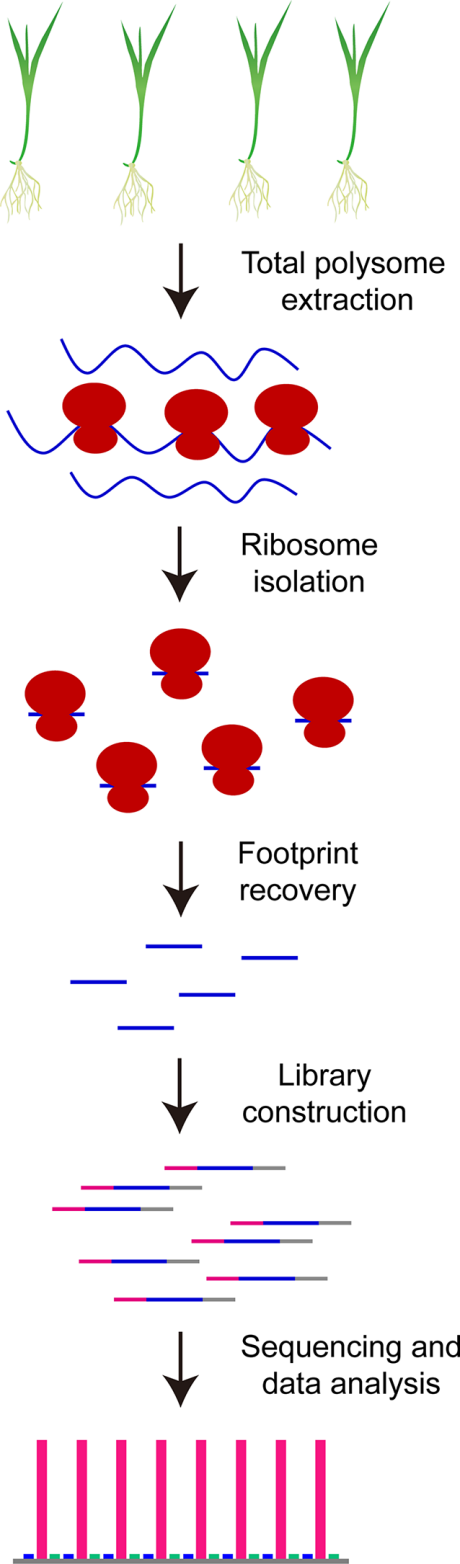
Schematic illustration for construction of high-quality rice ribosome footprint library.

Recently, a genome-wide methodology named as ribosome profiling or ribo-seq has been first proposed by Ingolia et al. (2009) to evaluate *in vivo* RNA translation dynamics in yeast *via* high-throughput sequencing of ribosome footprints, and subsequently been rapidly extended to related research in bacteria (Li et al., 2012; Mohammad et al., 2016; Mohammad et al., 2019), animals (Ingolia et al., 2011; Gonzalez et al., 2014; Chen and Dickman, 2017; Sugiyama et al., 2017; Zhang et al., 2018a), human being (Ingolia et al., 2014; Raj et al., 2016; Blair et al., 2017; Holmes et al., 2019; Chen et al., 2020) and higher plants (Liu et al., 2013; Juntawong et al., 2014; Lei et al., 2015; Merchante et al., 2015; Hsu et al., 2016; Bazin et al., 2017; Lorenzo-Orts et al., 2019; Wu H. L. et al., 2019). To perform ribosome profiling, total polysomes are first isolated from tissues of interest such as rice seedling shoots, then the monosomes that are derived from the nuclease-treated total polysomes are collected for isolation of ribosome footprints, which are subjected to rRNA trimming and library construction, and the final product is obtained for high-throughput sequencing followed by data analysis after PCR enrichment and PAGE purification of ribosome footprint library (**Figure 1**). Ribosome profiling results are thus able to provide the detailed information about ribosome occupancy on mRNAs at a given time in organisms, and as a result, our mechanistic understanding about the biological functions of ribosomes and translation regulation is greatly improved. For example, Michel and co-authors (2014) have investigated translation initiation of ribosomes at individual start codons in mammalian cells by using ribo-seq datasets and found the overwhelming preference of AUG at translation initiation sites (TIS) and downstream TIS; in contrast, the diverse contributions of different codons have been observed for upstream TIS including approximately 25% of AUG, approximately 30% of CUG and approximately 40% of AUG variants such as UUG, GUG, AGG, and ACG. The translation elongation speed and stalling have also been evaluated in bacteria, yeast and mammalian cells with ribo-seq results (Li et al., 2012; Li et al., 2019; Wu C. C. C. et al., 2019). Andreev et al. (2015) have investigated the translation responses of cultured human cells under sodium arsenite stress with ribosome profiling and found that upstream open reading frames (uORFs) hidden in 5’ leader sequences of genes resistant to eIF2 repression are widely translated and function as translation repressors for their downstream main ORFs (mORFs). Furthermore, some of these regulatory uORFs potentially encode functional protein products (Andreev et al., 2015). The functional uORFs have also been reported in yeast under starvation condition (Ingolia et al., 2009) and *Arabidopsis* under phosphate deficiency (Bazin et al., 2017). Ribosome profiling has been used to assess codon usage frequency over translation course in eukaryotes as well (Gorochowski et al., 2015).

In comparison to its widespread application in bacteria, yeast, animals and human being, only a limited number of studies on ribosome profiling have been conducted in higher plants (Liu et al., 2013; Juntawong et al., 2014; Lei et al., 2015; Merchante et al., 2015; Hsu et al., 2016; Bazin et al., 2017; Lorenzo-Orts et al., 2019; Wu H. L. et al., 2019), and most of them are restricted to the model plant *Arabidopsis*. Up to date, no available protocol has been proposed for construction of high-quality ribosome footprint library in rice, which feeds most of world population. Even for *Arabidopsis*, most of the current protocols for construction of ribosome footprint library are still challenged because, on one hand, the peak length of the yielded ribosome footprints displays large deviations from the canonical 28 nucleotides (nt), and on the other hand, the 3-nt periodicity of the yielded ribosome footprints, a critical feature for identification of novel ORFs, is very weak or insignificant (Liu et al., 2013; Juntawong et al., 2014; Lei et al., 2015; Merchante et al., 2015; Bazin et al., 2017). Although an improved protocol has been reported for construction of *Arabidopsis* ribosome footprint library in a recent study (Hsu et al., 2016), it remains largely unknown to what extents it could be applied to rice, because of the large divergence in many aspects such as genomic composition and plant texture between them. Thus to construct high-quality ribosome footprint library for both basic and applied research in rice is urgently needed.

In this study, a protocol for construction of high-quality rice ribosome footprint library is provided. Rice total polysomes are first isolated with a modified low ionic polysome extraction buffer. After nuclease digestion, rice ribosome footprints are extracted using SDS method followed by column purification. High-quality rice ribosome footprint library with peak reads of about 28 nt length and strong 3-nt periodicity, two crucial characteristics superior to most ribosome footprint libraries constructed in *Arabidopsis* previously (Liu et al., 2013; Juntawong et al., 2014; Merchante et al., 2015; Bazin et al., 2017), is prepared by following key steps of rRNA depletion, end repair, 3’ adapter ligation, reverse transcription, circularization, PCR enrichment and several rounds of purification. The generality of this protocol is confirmed by evaluating the length, 3-nt periodicity and gene body distribution of ribosome footprints in the libraries constructed with different rice cultivars under normal and salt stress conditions. Discordance between transcriptomes and translatomes was further revealed by comparing gene expression at transcription and translation levels in rice under salt stress, indicating that translation regulation represents an independent layer for rice acclimation to salt stress. In addition, different types of small ORFs were identified in rice genome by using the ribosome footprint libraries. Finally, we propose a series of key points, to which more attentions should be paid during library construction process.

## Materials and Methods

### Plant Materials, Growth Conditions, and Sample Collection

To evaluate the generality of this protocol, two rice (*Oryza sativa* L.) cultivars “Nipponbare” (NB) (ssp. *japonica*) and “Sea Rice 86” (SR86) (ssp. *indica*) under different growth conditions were used. In brief, the sterilized seeds of NB and SR86 were placed in a growth chamber, which was set as 28°C of air temperature without lighting, for germination. Then the germinated seedlings of NB and SR86 were grown in hydroponic boxes with Yoshida solution (Yoshida et al., 1976) in the growth chamber, which was set as 12 h of photoperiod, 28°C of air temperature for lighting period, 25°C of air temperature for dark period, and 65% ± 5% of air relative humidity. The refreshment of nutrition solution in hydroponic boxes was carried out every 4 days and 150 mM salt stress treatment was started for the seedlings of NB and SR86 at three-leaf stage. Seedling shoots of both rice cultivars were collected before (0 h) and after 24-h salt stress treatment (24 h), respectively. The sampled shoots were immediately frozen in liquid nitrogen and stored at −80 – −65°C until the commencement of library construction. Three biological repeats were prepared for NB and SR86 rice, respectively.

### Reagents, Kits, and Plastics

The reagents, kits and plastics used for construction of rice ribosome footprint library are listed as follow: tris base; hydrogen chloride (HCl); potassium chloride (KCl); magnesium chloride (MgCl_2_); polyoxyethylene (10) tridecyl ether (PTE); deoxycholic acid (DOC); DL-dithiothreitol (DTT); cycloheximide; DNase I; sucrose; SUPERase-in RNase inhibitor; ethanol; urea; 40% (W/V) Acrylamide/Bis; boron; ethylenediaminetetraacetic acid (EDTA); ammonium persulfate (APS); N,N,N’,N’-tetramethylethylenediamine (TEMED); 6 × Blue/Orange loading dye; SYBR Gold nucleic acid gel stain; glycogen; isopropanol; sodium chloride (NaCl); sodium acetate (NaAc); 0.45 μm COSTAR Spin-X filter; Agencourt AMPure XP beads; TruSeq Mammalian Ribo Profile Kit (ASLPA1212, Illumina); Ribo-Zero rRNA Removal Kit for Plant Leaf (MRZPL1224, Illumina); Zymo RNA clean and concentrator kit R1015; Zymo RNA clean and concentrator kit R1017; 1.5-mL, 2-mL and 15-mL DNase/RNase-free tube; 200-μL DNase/RNase-free PCR tube; polypropylene centrifuge tube, 13 mm × 51 mm; and Illustra MicroSpin S-400 HR column.

### Oligonucleotides

The sequences of oligonucleotides used for construction of rice ribosome footprint library are adopted from the manual of TruSeq Mammalian Ribo Profile Kit (ASLPA1212, Illumina) and listed in **Table 1**.

**Table 1:** The detailed information of DNA/RNA sequences in construction of rice ribosome footprint library.

DNA/RNA	Sequence
28-nt control RNA*	NNGUACACGGAGUCGACCCGCAACGCNN
30-nt control RNA*	NNGUACACGGAGUCAAGACCCGCAACGCNN
3’ adapter	AGATCGGAAGAGCACACGTCT
Forward primer	AATGATACGGCGACCACCGAGATCTACACGTTCAGAGTTCTACAGTCCGACG
Index primer 1**	CAAGCAGAAGACGGCATACGAGATATCACGGTGACTGGAGTTCAGACGTGTGCTCTTCCGATCT
Index primer 2**	CAAGCAGAAGACGGCATACGAGATCGATGTGTGACTGGAGTTCAGACGTGTGCTCTTCCGATCT
Index primer 3**	CAAGCAGAAGACGGCATACGAGATTTAGGCGTGACTGGAGTTCAGACGTGTGCTCTTCCGATCT
Index primer 4**	CAAGCAGAAGACGGCATACGAGATTGACCAGTGACTGGAGTTCAGACGTGTGCTCTTCCGATCT
Index primer 5**	CAAGCAGAAGACGGCATACGAGATACAGTGGTGACTGGAGTTCAGACGTGTGCTCTTCCGATCT
Index primer 6**	CAAGCAGAAGACGGCATACGAGATGCCAATGTGACTGGAGTTCAGACGTGTGCTCTTCCGATCT
Index primer 7**	CAAGCAGAAGACGGCATACGAGATCAGATCGTGACTGGAGTTCAGACGTGTGCTCTTCCGATCT
Index primer 8**	CAAGCAGAAGACGGCATACGAGATACTTGAGTGACTGGAGTTCAGACGTGTGCTCTTCCGATCT
Index primer 9**	CAAGCAGAAGACGGCATACGAGATGATCAGGTGACTGGAGTTCAGACGTGTGCTCTTCCGATCT
Index primer 10**	CAAGCAGAAGACGGCATACGAGATTAGCTTGTGACTGGAGTTCAGACGTGTGCTCT TCCGATCT
Index primer 11**	CAAGCAGAAGACGGCATACGAGATGGCTACGTGACTGGAGTTCAGACGTGTGCTCTTCCGATCT
Index primer 12**	CAAGCAGAAGACGGCATACGAGATCTTGTAGTGACTGGAGTTCAGACGTGTGCTCTTCCGATCT

* “N” represents A, or U, or C, or G; ** Index nucleotides in these primers are indicated in red.

### Equipment

Experimental equipment required for rice ribosome footprint library construction mainly includes a high-speed centrifuge for isolation of total polysomes, an ultracentrifuge for separation of total polysomes on a sucrose gradient, a gradient fractionator system with a UA absorbance detector for polysome profile analysis, a dry bath for incubation of nuclease-treated polysome samples, a spectrophotometer for determination of RNA concentration, a vertical electrophoresis system for PAGE purification, and a thermocycler for reverse transcription and PCR amplification.

### procedures

#### Preparation of Rice Total Polysomes

Rice tissue of interest is well ground to powder with mortar and pestle in liquid nitrogen. About 1 g of the powder is resuspended in 5 mL of ice-cold polysome extraction buffer (PEB) [100 mM Tris-HCl (pH 8.0), 40 mM KCl, 20 mM MgCl_2_, 2% (V/V) PTE (SIGMA), 0.2% (W/V) DOC (SIGMA), 1 mM DTT, 100 μg mL^−1^ cycloheximide (SIGMA) and 10 U mL^−1^ DNase I (Epicentre)]. The suspension is centrifuged at 5,000 g for 10 min at 4°C followed by another 10-min centrifugation in a new 15-mL DNase/RNase-free tube at 20,000 g at 4°C in Avanti J-E high-speed centrifuge (Beckman). RNA concentration and A260 unit of the supernatant are measured with a Nanodrop 2000 spectrophotometer (Thermo), and then used for polysome profile analysis and isolation of ribosome footprints.

#### Profile Analysis for Rice Polysomes

**Figure 2:**
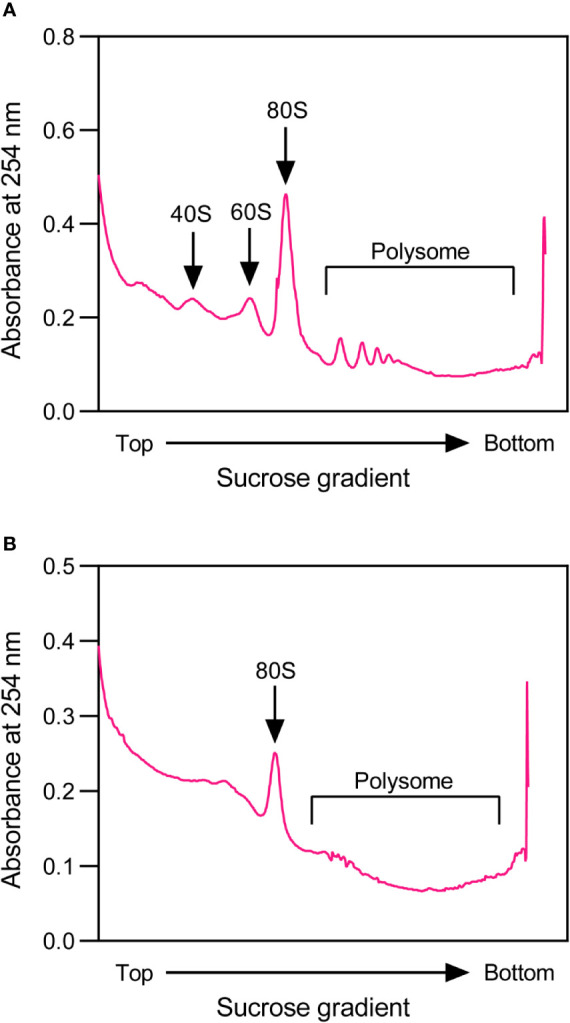
Representative profiles of rice total polysomes for construction of high-quality rice ribosome footprint library. **(A)** A representative profile of rice total polysomes before nuclease digestion. **(B)** A representative profile of rice total polysomes after nuclease digestion.

To perform profile analysis, 1,000 A260 units of the isolated rice total polysomes are loaded on a 15 – 60% (W/V) sucrose gradient that is prepared in a polypropylene centrifuge tube (13 mm × 51 mm, Beckman) using a peristaltic pump (BT101S, Lead Fluid). The sucrose gradient with polysome sample is then centrifuged in an SW-55 rotor (Beckman) at 170,000 g for 1.5 h at 4°C. Fractionation, ultraviolet absorbance assay at 254 nm and data acquisition of the resulting sample are performed using a gradient fractionator system (BRANDEL) with a UA-6 absorbance detector (Teledyne ISCO), and a representative polysome profile is shown in **Figure 2A**. The profile in rice is similar to that observed in *Arabidopsis* (Hsu et al., 2016).

#### Preparation of Rice Ribosome Footprints

For isolation of ribosome footprints, the RNA concentration of rice polysome extract is first adjusted to 400 ng μL^−1^, and then 200 μL aliquot is digested with the Illumina TruSeq Ribo Profile Nuclease (0.5 U μg^−1^ RNA) for approximately 1.4 h in a dry bath (Thermo) that is set at 25°C with shaking speed of 600 rpm. This digestion is stopped by adding 15 μL of SUPERase-in RNase inhibitor (Thermo) to the reaction mixture. 100 μL of digested lysate are aliquoted to perform profile analysis to evaluate if the nuclease digestion condition is optimal, and the representative polysome profile is shown in **Figure 2B**. After digestion, the polysome parts are almost gone, indicating that the digestion is sufficient. The ribosome footprints are then isolated immediately by loading the remaining digested lysate on an equilibrated Illustra MicroSpin S-400 HR column (GE Healthcare) with 3 mL of PEB, followed by spinning at room temperature (RT) with 600 rpm for 2 min. After adding 20 μL of 10% (W/V) SDS into the filtrate, RNAs are extracted using Zymo RNA clean and concentrator kit R1017 (Zymo Research), and further concentrated using R1015 (Zymo Research), according to the manufacturer’s manuals with minor modifications.

1. Extraction and purification of rice ribosome footprints by R1017 kit: Add two volumes of RNA Binding Buffer into the isolated ribosome footprints and mix well. Thereafter, one volume of 100% (V/V) ethanol is added into the mixture and mix well. The resulting solution is transferred to a column that is provided in the R1017 kit and centrifuged with 12,000 g at RT for 30 s. The filtrate is transferred to the same column and the centrifugation is repeated once more. Discard the filtrate and an on-column DNase I treatment is performed for the ribosome footprints that are bound in the column: the column is first washed by adding 400 μL of RNA Wash Buffer followed by centrifugation with 12,000 g for 30 s at RT; then 80 μL of DNase I treatment solution (5 μL DNase I + 75 μL DNase I Digestion Buffer) is loaded into the washed column and incubated at RT for 15 min to make sure that the DNAs are completely removed in the ribosome footprint sample. After 15-min incubation, the column is washed by 400 μL of RNA Pre Buffer with 12,000 g centrifugation at RT for 30 s, followed by 700 μL and 400 μL of RNA Wash Buffer with 12,000 g centrifugation at RT for 30 s and 2 min, respectively. Finally, the ribosome footprints bound by the column are eluted by 2-min incubation of the column with 50 μL RNase-free H_2_O and two rounds of 16,000 g centrifugation at RT for 1 min (safety stop point and the obtained ribosome footprint solution can be stored at −80 – –65°C).
2. Concentration of rice ribosome footprints by R1015 kit: Add two volumes of RNA Binding Buffer into the obtained ribosome footprints and mix well. Thereafter, one volume of 100% (V/V) ethanol is added into the mixture and mix well. The resulting solution is transferred to a column that is provided in the R1015 kit, and centrifuged with 12,000 g at RT for 30 s. The filtrate is transferred to the same column and the centrifugation is repeated once more. Discard the filtrate and the column is washed by 400 μL of RNA Pre Buffer with 12,000 g centrifugation at RT for 30 s, followed by 700 μL and 400 μL of RNA Wash Buffer with 12,000 g centrifugation at RT for 30 s and 2 min, respectively. Finally, the ribosome footprints bound by the column are eluted by 2-min incubation of the column with 26 μL RNase-free H_2_O and two rounds of 16,000 g centrifugation at RT for 1 min (safety stop point and the obtained ribosome footprint solution can be stored at −80 – −65°C).

#### rRNA Depletion in Rice Ribosome Footprints

rRNA depletion and recovery of rice ribosome footprints, of which most are around 28 – 30 nt, are performed with the Ribo-Zero rRNA Removal Kit for Plant Leaf (MRZPL1224, Illumina) and PAGE purification, respectively. The detail for rRNA depletion and footprint purification is as follow.

1. Preparation of rRNA depletion beads: Transfer 225 μL of rRNA depletion magnetic beads to a 1.5-mL DNase/RNase-free tube and place the tube on a magnetic stand until the liquid in the tube becomes clear. Discard the supernatant, add 225 μL RNase-free H_2_O into the tube to resuspend the rRNA depletion magnetic beads by vortex. Then the tube is placed on the magnetic stand until the mixture becomes clear and discard the supernatant. The rRNA depletion magnetic beads are washed again by 225 μL of RNase-free H_2_O and the resulting beads are finally resuspended in 65 μL of Magnetic Bead Resuspension Solution.
2. Hybridization of probes with rRNAs in rice ribosome footprints: Prepare hybridization mixture in a 200-μL DNase/RNase-free PCR tube according to **Table 2**, pipette the solution well and place the tube in a thermocycler (Bio-Rad) that is set at 68°C for 10 min. Thereafter the tube is centrifuged shortly at low speed and incubated at RT for 5 min.
3. rRNA depletion: Transfer the solution obtained from the step (2) to the tube that contains 65 μL of rRNA depletion magnetic beads from the step (1), pipette the mixture well and incubate it at RT for 10 min. Place the tube on the magnetic stand until the liquid becomes clear and then transfer the supernatant to a new 1.5-mL DNase/RNase-free tube. Keep the rRNA-depleted ribosome footprints on ice (safety stop point and the rRNA-depleted ribosome footprints can be stored at −25 – −15°C overnight or at −80 – –65°C for one month).
4. Purification of rice ribosome footprints by R1015 kit: The rRNA-depleted ribosome footprints are purified and recovered with Zymo RNA clean and concentrator kit R1015 according to the manufacturer’s manuals with minor modifications: make the volume of rRNA-depleted ribosome footprint solution to 100 μL by adding RNase-free H_2_O; then add 200 μL of RNA Binding Buffer and 450 μL of 100% (V/V) ethanol to the 100 μL rRNA-depleted footprint solution and mix well; transfer the resulting mixture to a column that is provided in the R1015 kit and centrifuge it with 12,000 g at RT for 30 s. The obtained filtrate is transferred to the same column and the centrifugation is repeated once more. Discard the filtrate and the column is washed by 400 μL of RNA Pre Buffer with 12,000 g centrifugation at RT for 30 s, followed by 700 μL and 400 μL of RNA Wash Buffer with 12,000 g centrifugation at RT for 30 s and 2 min, respectively. Finally, the ribosome footprints bound in the column are eluted by 2-min incubation of the column with 11 μL RNase-free H_2_O and two rounds of 16,000 g centrifugation at RT for 1 min.
5. PAGE purification of rice ribosome footprints: Prepare 15 mL of 1.00 mm 15% (W/V) urea-PAGE [6.3 g urea (SIGMA)、5.625 mL 40% (W/V) Acrylamide/Bis solution (Ambion), 0.75 ml 10 × TBE buffer, 12 μL 10% (W/V) APS (SIGMA) solution and 9 μL TEMED (SIGMA)]. Add 1 μL of 6 × Blue/Orange loading dye (Promega) to 5 μL of control RNAs (oligonucleotides of 28 nt and 30 nt) that are provided in the TruSeq Mammalian Ribo Profile Kit (Illumina), and 2 μL of 6 × Blue/Orange loading dye to the ribosome footprint solution (approximately 10 μL) from the step (4), respectively. Mix the control RNA and footprint samples well, and keep them on ice immediately after being treated in the dry bath at 95°C for 5 min. The cooled control and ribosome footprint samples are then separated in the 15% urea-PAGE by electrophoresis. The urea-PAGE is stained by pre-cooled SYBR Gold (Thermo) nucleic acid staining solution at 4°C for 10 min when the bromophenol blue reaches the bottom of gel. Gel slices that contain control RNAs and around 28 – 30 nt ribosome footprints are recovered under a dark-field transilluminator (TGreen) and transferred to 2-mL DNase/RNase-free tubes (**Supplementary Figure S1**). The gel slices are well ground and resuspended by adding 2 μL of 10% (W/V) SDS, 40 μL of 5 M acetic ammonium and 400 μL of RNase-free H_2_O. Place the 2-mL tubes on a Tube Revolver (MIULAB), and elute the control RNAs and ribosome footprints from the gel slices by continuous rotation with 30 rpm at 4°C overnight. Thereafter, transfer the liquids from the 2-mL DNase/RNase-free tubes to 0.45-μm COSTAR Spin-X filters (Corning) and centrifuge them with 12,000 g at RT for 10 min. Add 2 uL of glycogen (Invitrogen) and 700 μL isopropanol into each filtrate, mix well and then keep the filtrates at −25 – −15°C overnight. After centrifugation with 16,000 g at 4°C for 30 min – 1 h, the pellets of control and ribosome footprint samples are washed with 80% (V/V) ethanol, air-dried and finally resuspended in 8 uL and 20 uL of RNase-free H_2_O, respectively.

**Table 2:** Hybridization mixture.

Component	Weight/Volume
Ribosome footprint RNAs	5 μg in 26 μL
Ribo-Zero Removal Solution	10 μL
Ribo-Zero Reaction Buffer	4 μL
Total	40 μL

#### End Repair for Rice Ribosome Footprints

Prepare reaction mixture for end repair in a 200 μL DNase/RNase-free PCR tube based on **Table 3**. Pipette the mixture well and place the tube in the thermocycler that is set at 37°C for 1 h. The end-repaired ribosome footprints are purified and recovered with Zymo RNA clean and concentrator kit R1015: make the volume of end-repaired ribosome footprint solution to 100 μL by adding RNase-free H_2_O; then add 200 μL of RNA Binding Buffer and 450 μL of 100% (V/V) ethanol to the 100 μL end-repaired footprint solution and mix well; transfer the resulting liquid to a column that is provided in the R1015 kit and centrifuge it with 12,000 g at RT for 30 s. The obtained filtrate is transferred to the same column and the centrifugation is repeated once more. Discard the filtrate and the column is washed by 400 μL of RNA Pre Buffer with 12,000 g centrifugation at RT for 30 s, followed by 700 µL and 400 μL of RNA Wash Buffer with 12,000 g centrifugation at RT for 30 s and 2 min, respectively. Finally, the ribosome footprints bound in the column are eluted by 2-min incubation of the column with 11 μL RNase-free H_2_O and two rounds of 16,000 g centrifugation at RT for 1 min. Place the recovered ribosome footprint sample on ice for subsequent 3’ adapter ligation.

**Table 3:** Reaction mixture for end repair.

Component	Volume
Ribosome footprints	20 uL
TruSeq Ribo PNK Buffer	7.5 uL
TruSeq Ribo PNK	3 uL
RNase-free H_2_O	44.5 uL
Total	75 uL

#### 3’ Adapter Ligation for Repaired Rice Ribosome Footprints

Add 1 μL of TruSeq Ribo Profile 3’ Adapter into the control RNA and end-repaired ribosome footprint samples, respectively, and then place these samples in the thermocycler that is set at 65°C for 2 min followed by 4°C for ever. Thereafter, 3.5 μL of TruSeq Ribo Profile Ligation Buffer, 1 μL of 100 mM DTT and 1.5 μL of TruSeq Ribo Profile Ligase are added into both the control and ribosome footprint samples, mixed well and then placed in the thermocycler that is set at 23°C for 2 h. Finally, 2 μL of TruSeq Ribo Profile AR Enzyme is added into both the control and ribosome footprint samples, mixed well and kept at 30°C for 2 h in the thermocycler. The resulting samples are placed on ice.

#### Reverse Transcription

Add 4.5 μL of TruSeq Ribo Profile RT Reaction Mix, 1.5 μL of 100 mM DTT, 1 μL of EpiScript RT and 6 μL of RNase-free H_2_O into both the control and ribosome footprint samples, mix well and then keep them at 50°C for 30 min in the thermocycler. Thereafter, 1 μL of TruSeq Ribo Profile Exonuclease is added into the control and ribosome footprint samples, mixed well and kept at 37°C for 30 min, at 80°C for 15 min and at 4°C for ever in the thermocycler (safety stop point and the obtained control and ribosome footprint libraries can be kept at −20°C or lower temperature for long-term storage). Thereafter, add 1 μL of TruSeq Ribo Profile RNase Mix into the control and ribosome footprint libraries, mix well and place these samples in the thermocycler that is set at 55°C for 5 min to remove the remaining RNAs in the libraries.

#### Purification of Rice Ribosome Footprint Library

##### Purification of Library by R1015 Kit

Make the volume of control and ribosome footprint libraries to 50 μL by adding RNase-free H_2_O; then add 100 μL of RNA Binding Buffer and 150 μL of 100% (V/V) ethanol to both the 50 μL control and ribosome footprint libraries. Mix the libraries well, transfer the resulting liquids to columns that are provided in the R1015 kit, and centrifuge them with 12,000 g at RT for 30 s. The obtained filtrates are transferred to the same columns and the centrifugation is performed once more. Discard the filtrates and wash the columns by 400 μL of RNA Pre Buffer with 12,000 g centrifugation at RT for 30 s, followed by 700 μL and 400 μL of RNA Wash Buffer with 12,000 g centrifugation at RT for 30 s and 2 min, respectively. Finally, the control and ribosome footprint libraries bound in the columns are eluted by 2-min incubation of the columns with 11 μL RNase-free H_2_O and two rounds of 16,000 g centrifugation at RT for 1 min. Place the recovered libraries on ice.

##### Purification of Library by 10% Urea-PAGE

Prepare 15 mL of 1.00 mm 10% (W/V) urea-PAGE [6.3 g urea, 3.75 mL 40% (W/V) Acrylamide/Bis solution, 0.75 ml 10 × TBE buffer, 12 μL 10% (W/V) APS solution and 9 μL TEMED]. Add 2 μL of 6 × Blue/Orange loading dye to both the control and ribosome footprint libraries (approximately 10 μL). Mix the libraries well and then keep them on ice immediately after being treated in the dry bath at 95°C for 5 min. The cooled control and ribosome footprint libraries are then separated in the 10% urea-PAGE by electrophoresis. The urea-PAGE is stained by pre-cooled SYBR Gold nucleic acid staining solution at 4°C for 10 min when the bromophenol blue migrates out of gel. Gel slices that contain 70 – 90 nt control and ribosome footprint libraries are recovered under the dark-field transilluminator, and then transferred to 2-mL DNase/RNase-free tubes (**Supplementary Figure S2**). The gel slices are well ground and resuspended by adding 2 μL of 10% (W/V) SDS, 40 μL of 5 M acetic ammonium and 400 μL of RNase-free H_2_O. Place the 2-mL tubes on the Tube Revolver, and elute the control and ribosome footprint libraries from the gel slices by continuous rotation with 30 rpm at 4°C overnight. Thereafter, transfer the mixture from the 2-mL tubes to 0.45-μm COSTAR Spin-X filters and centrifuge them with 12,000 g at RT for 10 min. Add 2 uL of glycogen and 700 μL of isopropanol into the filtrates, mix well and then keep the filtrates at −25 – −15°C overnight. After 16,000 g centrifugation at 4°C for 30 min ~ 1 h, the pellets of control and ribosome footprint libraries are washed with 80% (V/V) ethanol, air-dried and finally resuspended in 10 uL nuclease-free H_2_O, respectively (safety stop point and all library samples can be stored at −20°C or lower temperature for a long term).

#### Circularization and PCR Enrichment for Rice Ribosome Footprint Library

Prepare circularization reaction mixture based on **Table 4** and pipette it well. The mixture is kept in a thermocycler at 60°C for 2 h and then ice-cooled. Prepare PCR enrichment mixture according to **Table 5**, pipette the mixture well and then start the enrichment reaction based on the following PCR procedure: 98°C for 30 s; 94°C for 15 s, 55°C for 5 s, 65°C for 10 s, 9 – 13 cycles; 4°C for ever.

**Table 4:** Reaction mixture for circularization.

Component	Volume
cDNAs	10 μL
TruSeq Ribo Profile CL Reaction Mix	4 μL
ATP	2 μL
MnCl_2_	2 μL
CircLigase	2 μL
Total	20 μL

**Table 5:** PCR reaction mixture for library enrichment.

Component	Volume
Circularized cDNAs	5 μL
TruSeq Ribo Profile Forward PCR Primer	2 μL
TruSeq Ribo Profile Index PCR Primer	2 μL
2 × Phusion MasterMix	25 μL
Nuclease-free H_2_O	16 μL
Total	50 μL

#### Purification of Enriched Rice Ribosome Footprint Library

##### Purification of Enriched Rice Ribosome Footprint Library by Agencourt AMPure XP Beads

Transfer the enriched control and ribosome footprint libraries to 90 μL of Agencourt AMPure XP beads (Beckman) in 1.5-mL DNase/RNase-free tubes, pipette the mixture well and place the tubes on the magnetic stand until the liquid becomes clear. Discard the supernatant, resuspend the beads in 200 μL of 70% (V/V) ethanol by pipetting and then keep the tubes at RT for 5 min. Place the tubes on the magnetic stand until the mixture becomes clear. Discard supernatant and repeat the washing. The double-washed beads are air-dried and resuspended with 16 μL nuclease-free H_2_O. Place the tubes on the magnetic stand until the liquid becomes clear. Transfer the supernatant into new 1.5-mL DNase/RNase-free tubes and place the tubes on ice.

##### Purification of Enriched Rice Ribosome Footprint Library by 8% Native PAGE

Prepare 15 mL of 1.00 mm 8% (W/V) native PAGE [3 mL 40% (W/V) Acrylamide/Bis solution, 0.75 ml 10 × TBE buffer, 12 μL 10% (W/V) APS solution and 9 μL TEMED]. Add 3 μL of 6 × Blue/Orange loading dye into both the enriched control and ribosome footprint libraries (approximately 15 μL). Mix the enriched libraries well and then separate them in the 8% native PAGE by electrophoresis. The native PAGE is stained by pre-cooled SYBR Gold nucleic acid staining solution at 4°C for 10 min when the bromophenol blue reaches the bottom of gel. Gel slice that contains 140 – 160 nt enriched ribosome footprint library is recovered under dark-field transilluminator and transferred to a 2-mL DNase/RNase-free tube (**Supplementary Figure S3**). The gel slice is well ground and resuspended by adding 400 μL of 0.4 M NaCl solution. Place the 2-mL tube on the Tube Revolver and elute the enriched ribosome footprint library from the gel slice by continuous rotation with 30 rpm at 4°C overnight. Thereafter, transfer the mixture from the 2-mL tube to a 0.45-μm COSTAR Spin-X filter and centrifuge it with 12,000 g at RT for 10 min. Add 2 uL of glycogen, 40 μL of 3 M NaAc (pH 5.2) and 1 mL of 100% (V/V) ethanol into the filtrate, mix well and then keep the filtrate at −80 – −65°C overnight. After 16,000 g centrifugation at 4°C for 30 min – 1 h, the pellet of enriched ribosome footprint library is washed with 80% ethanol, air-dried and finally resuspended in 15 uL of nuclease-free H_2_O for subsequent sequencing analysis.

#### High-Throughput Sequencing of Rice Ribosome Footprint Library and Data Analysis

**Figure 3:**
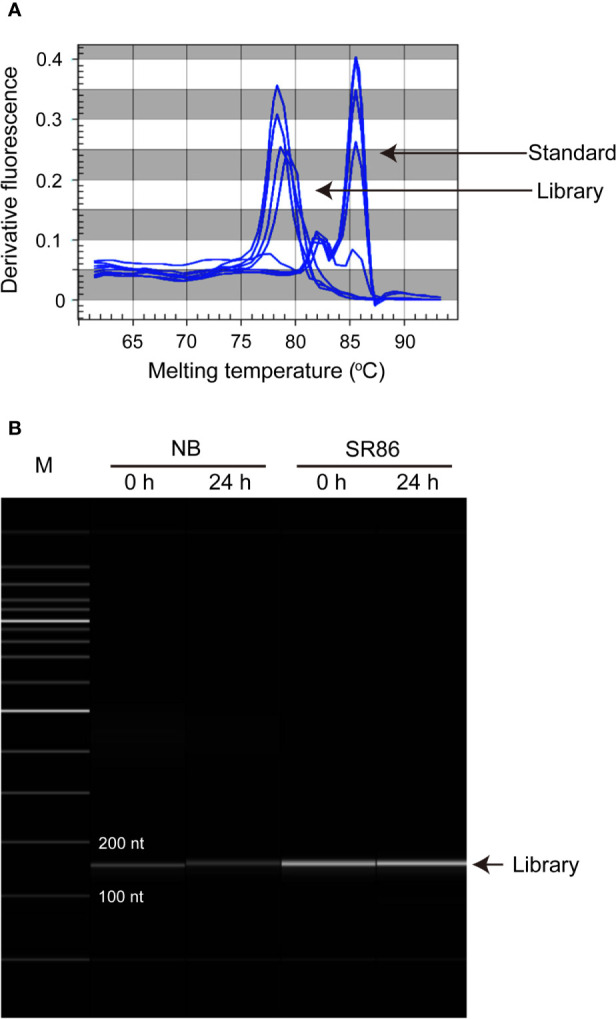
Quantification and qualification of rice ribosome footprint libraries before high-throughput sequencing. **(A)** Quantification and qualification of the rice ribosome footprint libraries constructed with seedling shoots of “Nipponbare” (NB) and “Sea Rice 86” (SR86) under normal growth condition (0 h) and after 24-h salt stress treatment (24 h) by qPCR. **(B)** Quantification and qualification of the rice ribosome footprint libraries constructed with seedling shoots of NB and SR86 under normal growth condition (0 h) and after 24-h salt stress treatment (24 h) by capillary electrophoresis.

The obtained rice ribosome footprint library is subjected to concentration determination and length checking by qPCR and capillary electrophoresis (**Figure 3**), respectively, followed by high-throughput sequencing with a given strategy such as the single-end 50 bp on a HiSeq2500 platform. Bioinformatics analysis, briefly including adapter removal, quality assessment, mapping to a reference genome, normalization and calculation of gene expression, will then be applied for the raw data of rice ribosome footprint library with the aid of pipelines such as RiboProfiling under R environment (Popa et al., 2016), RiboTraper under Unix environment (Calviello et al., 2016), or RiboCode under Python environment (Xiao et al., 2018).

## Results and Discussion

### Quality Evaluation of Rice Ribosome Footprint Library

By following the abovementioned procedures, we constructed ribosome footprint libraries with seedling shoots of two rice cultivars, NB and SR86, which were grown under normal and salt stress conditions respectively. Three biological repeats were performed for each cultivar. With the aid of RiboCode pipeline (Xiao et al., 2018), the quality of these rice ribosome footprint libraries was evaluated by examining the size, 3-nt periodicity and gene body distribution of the ribosome footprints.

**Figure 4:**
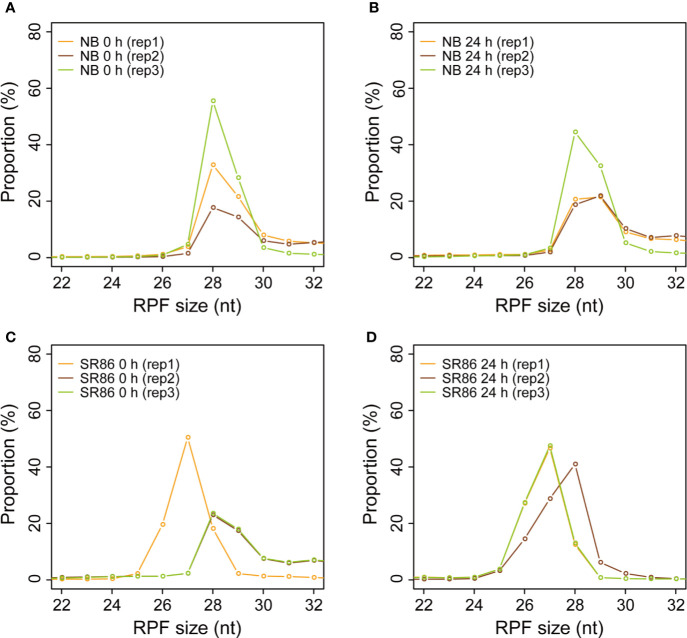
Size distribution of ribosome footprints in various rice ribosome footprint libraries. **(A)** Size distribution of ribosome footprints in the rice ribosome footprint libraries constructed with seedling shoots of “Nipponbare” (NB) under normal growth condition (0 h). **(B)** Size distribution of ribosome footprints in the rice ribosome footprint libraries constructed with seedling shoots of NB after 24-h salt stress treatment (24 h). **(C)** Size distribution of ribosome footprints in the rice ribosome footprint libraries constructed with seedling shoots of “Sea Rice 86” (SR86) under normal growth condition (0 h). **(D)** Size distribution of ribosome footprints in the rice ribosome footprint libraries constructed with seedling shoots of SR86 after 24-h salt stress treatment (24 h). “RPF” is short for “ribosome protected mRNA fragments”. RPF size is in nucleotide (nt). “rep1”, “rep2” and “rep3” represent the three biological repeats.

Canonically, the length of ribosome footprint is determined as 28 nt in organisms (Ingolia et al., 2009; Bazzini et al., 2014; Hsu et al., 2016; Wu H. L. et al., 2019). From our results, the size of rice ribosome footprints in all 12 libraries ranged from 26 nt to 30 nt, with that of 7 samples (NB 0 h rep1, NB 0 h rep2, NB 0 h rep3, NB 24 h rep3, SR86 0 h rep2, SR86 0 h rep3, and SR86 24 h rep2) peaking at 28 nt, 3 samples (SR86 0 h rep1, SR86 24 h rep1, and SR86 24 h rep3) peaking at 27 nt and 2 samples (NB 24 h rep 1, and NB 24 h rep2) peaking at 29 nt (**Figure 4**). Obviously, the majority of rice ribosome footprint libraries constructed with our protocol generated footprints with the canonical size.

**Figure 5:**
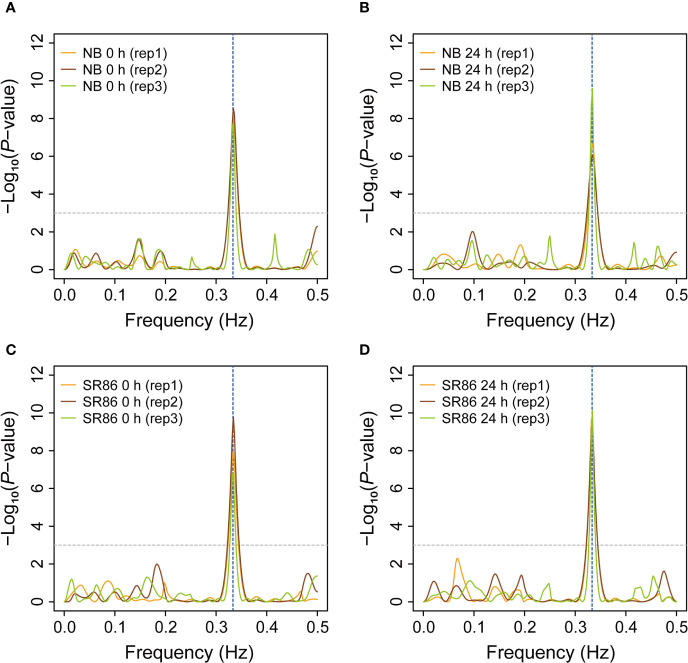
Periodicity of ribosome footprints in various rice ribosome footprint libraries. **(A)** Periodicity of ribosome footprints in the rice ribosome footprint libraries constructed with seedling shoots of “Nipponbare” (NB) under normal growth condition (0 h). **(B)** Periodicity of ribosome footprints in the rice ribosome footprint libraries constructed with seedling shoots of NB after 24-h salt stress treatment (24 h). **(C)** Periodicity of ribosome footprints in the rice ribosome footprint libraries constructed with seedling shoots of “Sea Rice 86” (SR86) under normal growth condition (0 h). **(D)** Periodicity of ribosome footprints in the rice ribosome footprint libraries constructed with seedling shoots of SR86 after 24-h salt stress treatment (24 h). The periodicity (in Hz) of rice ribosome footprints was evaluated by the F-score test implemented in “multitaper”, an R package. The horizontal dashed line indicates the cutoff (*P*-value = 0.001) for significant periodicity and the vertical dashed line shows the position of 1/3, the expected frequency (3-nt periodicity) of rice ribosome footprints. “rep1”, “rep2” and “rep3” represent the three biological repeats.

The 3-nt periodicity of ribosome footprints is a critical parameter for evaluation of library quality and identification of novel ORFs that are usually ignored during normal annotation process (Hsu et al., 2016; Wu H. L. et al., 2019). In the past several years, some ribosome footprint libraries have been reported in plants such as *Arabidopsis*, maize and tomato (Liu et al., 2013; Juntawong et al., 2014; Lei et al., 2015; Merchante et al., 2015; Hsu et al., 2016; Bazin et al., 2017; Lorenzo-Orts et al., 2019; Wu H. L. et al., 2019), while most of them generated ribosome footprints with weak or insignificant 3-nt periodicity (Liu et al., 2013; Juntawong et al., 2014; Lei et al., 2015; Merchante et al., 2015; Bazin et al., 2017). We wondered whether rice ribosome footprints generated from our protocol displayed significant 3-nt periodicity. To answer this question, we extracted footprints of peak size classes from the 12 rice ribo-seq libraries and evaluated their 3-nt periodicity by the F-score test implemented in an R package “multitaper” (Thomson, 1982), which can infer the periodicity and frequency of waves. The canonical ribosome footprints were expected to appear periodically on the transcripts with an interval of 3 nt, just resembling a wave with a periodicity of 1/3 Hz. As expected, we found that the extracted ribosome footprints displayed a strong 3-nt periodicity (*P*-value <= 0.001), though the peak size varied from 27 nt to 29 nt in the 12 rice ribosome footprint libraries (**Figure 5**).

**Figure 6:**
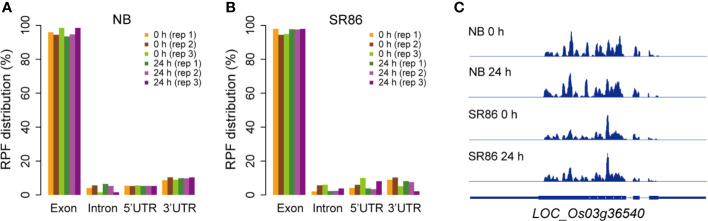
Percentage distribution of ribosome footprints on gene body in various rice ribosome footprint libraries. **(A)** Percentage distribution of ribosome footprints on exon, intron, 5’ UTR and 3’ UTR in the rice ribosome footprint libraries constructed with seedling shoots of “Nipponbare” (NB) under normal growth condition (0 h) and after 24-h salt stress treatment (24 h), respectively. **(B)** Percentage distribution of ribosome footprints on exon, intron, 5’ UTR and 3’ UTR in the rice ribosome footprint libraries constructed with seedling shoots of “Sea Rice 86” (SR86) under normal growth condition (0 h) and after 24-h salt stress treatment (24 h), respectively. “UTR” is short for “untranslated region”. “rep1”, “rep2” and “rep3” represent the three biological repeats. **(C)** Ribosome footprint coverage for *LOC_Os03g36540* in the rice ribosome footprint libraries constructed with seedling shoots of NB and SR86 under normal growth condition (0 h) and after 24-h salt stress treatment (24 h), respectively. The gene model for *LOC_Os03g36540* is provide at the bottom of ribo-seq panels, and the filled thinner rectangles, thicker rectangles and lines in blue represent UTR, exon and intron regions, respectively.

Previous studies have reported that, in contrast to RNA-seq libraries wherein a relatively high percentage of reads can be mapped to non-coding sequence (CDS) regions, the overwhelming majority of ribosome footprints in ribo-seq libraries are mapped to the exon regions with a very small proportion being mapped to introns and untranslated regions (UTRs) (Hsu et al., 2016; Wu H. L. et al., 2019). We thus analyzed the distribution of rice ribosome footprints, which were yielded by our protocol, on different genic elements including exon, intron, 5’ UTR and 3’ UTR regions. The results showed that the ribosome footprints in all 12 rice ribo-seq libraries were predominantly mapped to the exon regions, as opposed to the intron and UTR regions (**Figures 6A, B**). As a representative, *LOC_Os03g36540* was selected for displaying the ribosome footprint coverage on gene body in rice genome, and the results showed that the ribosome footprints were predominantly mapped to the exon regions with very few on the intron and UTR regions in the rice ribo-seq libraries, which were constructed with seedling shoots of both rice cultivars under normal and salt stress conditions (**Figure 6C**).

Altogether, the three anticipated features not only indicate that high-quality rice ribosome footprint libraries are successfully constructed but also well demonstrate the generality of this protocol.

### Discordance Between Transcriptomic and Translatomic Changes in Rice Under Salt Stress

**Figure 7:**
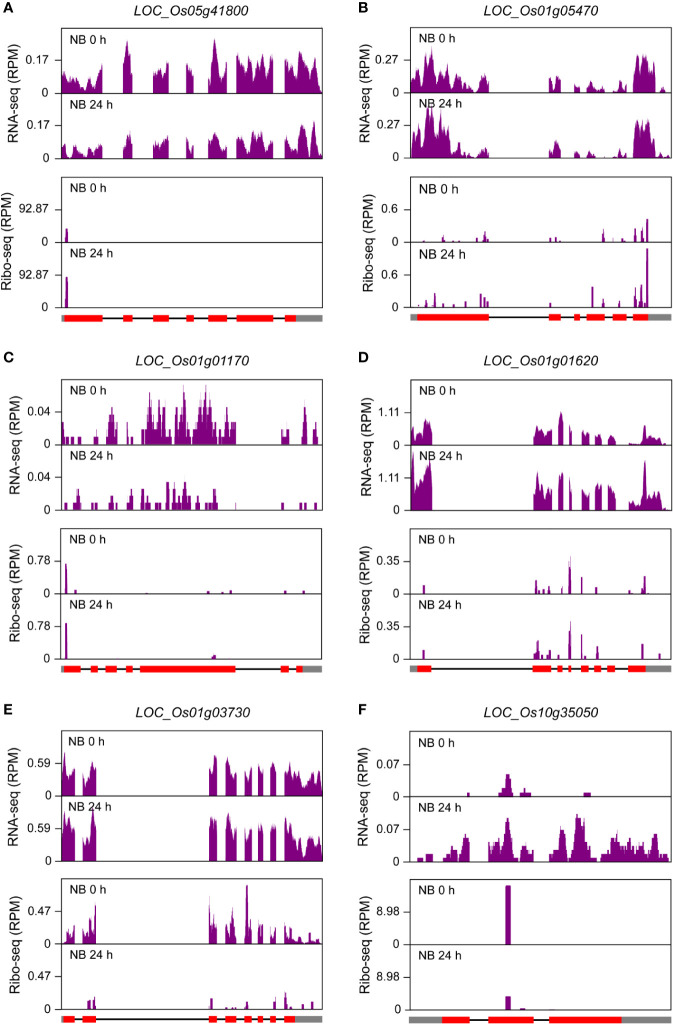
Discordance of differentially expressed genes (DEGs) at transcription and translation levels in seedling shoots of “Nipponbare” (NB) under salt stress. **(A)** RNA-seq and ribo-seq coverage for a representative (*LOC_Os05g41800*) of Group I DEGs (genes transcriptionally down-regulated but translationally up-regulated) in seedling shoots of NB under normal growth condition (0 h) and after 24-h salt stress treatment (24 h). **(B)** RNA-seq and ribo-seq coverage for a representative (*LOC_Os01g05470*) of Group II DEGs (genes only translationally up-regulated) in seedling shoots of NB under normal growth condition (0 h) and after 24-h salt stress treatment (24 h). **(C)** RNA-seq and ribo-seq coverage for a representative (*LOC_Os01g01170*) of Group III DEGs (genes only transcriptionally down-regulated) in seedling shoots of NB under normal growth condition (0 h) and after 24-h salt stress treatment (24 h). **(D)** RNA-seq and ribo-seq coverage for a representative (*LOC_Os01g01620*) of Group IV DEGs (genes only transcriptionally up-regulated) in seedling shoots of NB under normal growth condition (0 h) and after 24-h salt stress treatment (24 h). **(E)** RNA-seq and ribo-seq coverage for a representative (*LOC_Os01g03730*) of Group V DEGs (genes only translationally down-regulated) in seedling shoots of NB under normal growth condition (0 h) and after 24-h salt stress treatment (24 h). **(F)** RNA-seq and ribo-seq coverage for a representative (*LOC_Os10g35050*) of Group VI DEGs (genes transcriptionally up-regulated but translationally down-regulated) in seedling shoots of NB under normal growth condition (0 h) and after 24-h salt stress treatment (24 h). The cutoff values for DEGs are fold change >= 1.5 and *P*-value <= 0.01. “RPM” is short for “reads per million”. The filled rectangles in gray and red, and the black lines in gene models that are provided at the bottom of ribo-seq panels represent untranslated regions (UTRs), exons and introns, respectively.

**Figure 8:**
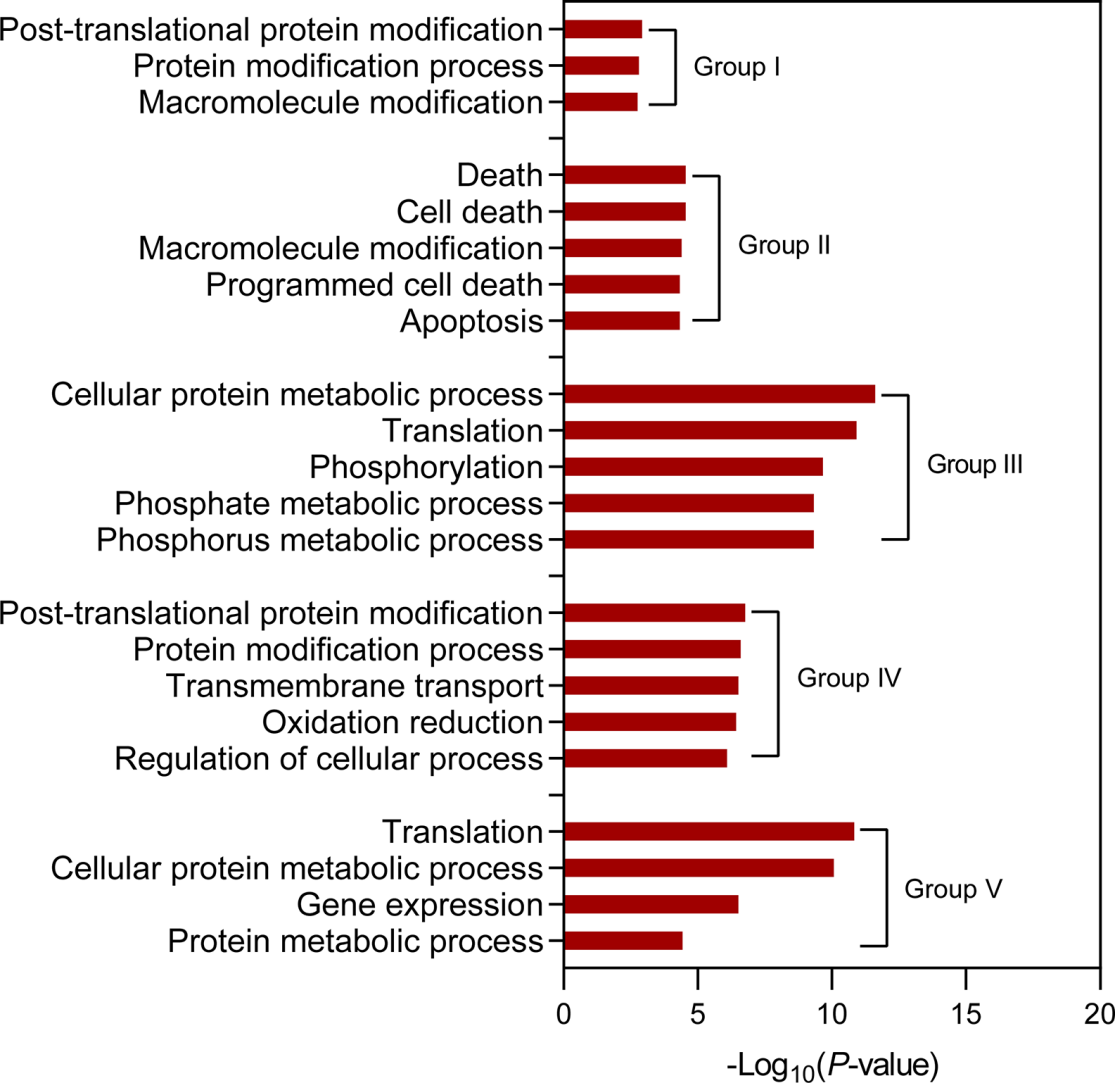
Identification of overrepresented gene ontology (GO) terms for the discordantly expressed genes at transcription and translation levels in seedling shoots of “Nopponbare” (NB) under salt stress. The cutoff value for the overrepresented GO terms is *FDR* <= 0.05. The top five overrepresented GO terms are displayed for the discordantly expressed genes of “Group II”, “Group III” and “Group IV”. “Group I”, “Group II”, “Group III”, “Group IV” and “Group V” represent “genes transcriptionally down-regulated but translationally up-regulated”, “genes only translationally up-regulated”, “genes only transcriptionally down-regulated”, “genes only transcriptionally up-regulated”, and “genes only translationally down-regulated”, respectively.

To investigate the relationship of transcription and translation responses to salt stress, we constructed RNA-seq libraries with samples from NB seedling shoots, which were same as those for the construction of ribosome footprint libraries, under normal and salt stress conditions. The fold changes of NB RNA-seq and ribo-seq signals were then compared. As expected, a large proportion of genes that were transcriptionally up-regulated or down-regulated simultaneously displayed significant stimulation or inhibition of their expression at translation level (fold change >= 1.5 and *P*-value <= 0.01), for example *LOC_Os01g03390* and *LOC_Os01g01120* (**Supplementary Figure S4**). Intriguingly, a number of genes were identified to be regulated discordantly at transcription and translation levels (fold change >= 1.5 and *P*-value <= 0.01) and categorized into six groups: I) genes transcriptionally down-regulated but translationally up-regulated, II) genes only translationally up-regulated, III) genes only transcriptionally down-regulated, IV) genes only transcriptionally up-regulated, V) genes only translationally down-regulated, and VI) genes transcriptionally up-regulated but translationally down-regulated (**Supplementary Table S1**). The representative examples for each group were shown in **Figure 7** and **Figure S5**. Genes belonging to Group III and Group IV are often considered as those that may undergo changes in translation subsequently if the stress condition persists (Lei et al., 2015; Lee et al., 2016), while genes of Group II and Group V reflect that they could respond to stress conditions more rapidly at translation level than at transcription level (Lei et al., 2015). There were a small portion of genes that were transcriptionally up-regulated but translationally down-regulated (**Figure 7F**; **Supplementary Table S1** and **Figure S5F**) possibly due to stimulated transcription coupled with the sequestration of translationally stalled mRNAs into stress granules (Koritzinsky et al., 2006). This kind of mRNAs could serve as mRNA reserves and undergo translation when stress conditions are relieved or eliminated (Parker and Sheth, 2007). Gene ontology (GO) term analysis for the genes of each group was further conducted and overrepresented GO terms were identified (**Figure 8**; **Supplementary Table S1**). The observed largely discordant regulation between transcriptomes and translatomes in rice under salt stress was consistent with previous findings that gene expression is controlled in a relatively independent manner at transcription and translation levels in *Arabidopsis* under hypoxia (Juntawong et al., 2014) and phosphate deficiency conditions (Bazin et al., 2017) and in maize under drought stress (Lei et al., 2015). The differentially translated genes from the ribosome footprint libraries were further confirmed by protein sequencing (fold change >= 1.5 and *P*-value <= 0.01), which was carried out with seedling shoots of NB under salt stress. For example, *LOC_Os01g42860*, *LOC_Os02g15860* and *LOC_Os02g44870*, three representative genes that were translationally up-regulated, displayed the increased protein abundance in response to salt stress (**Supplementary Figures S6A–C**); meanwhile, the expression of *LOC_Os02g57290*, *LOC_Os04g58200* and *LOC_Os12g08760*, three representative genes displaying down-regulated translation, were significantly decreased at protein level (**Supplementary Figures S6D–F**).

### Identification of Novel Open Reading Frames (ORFs) in Rice Genome

**Figure 9:**
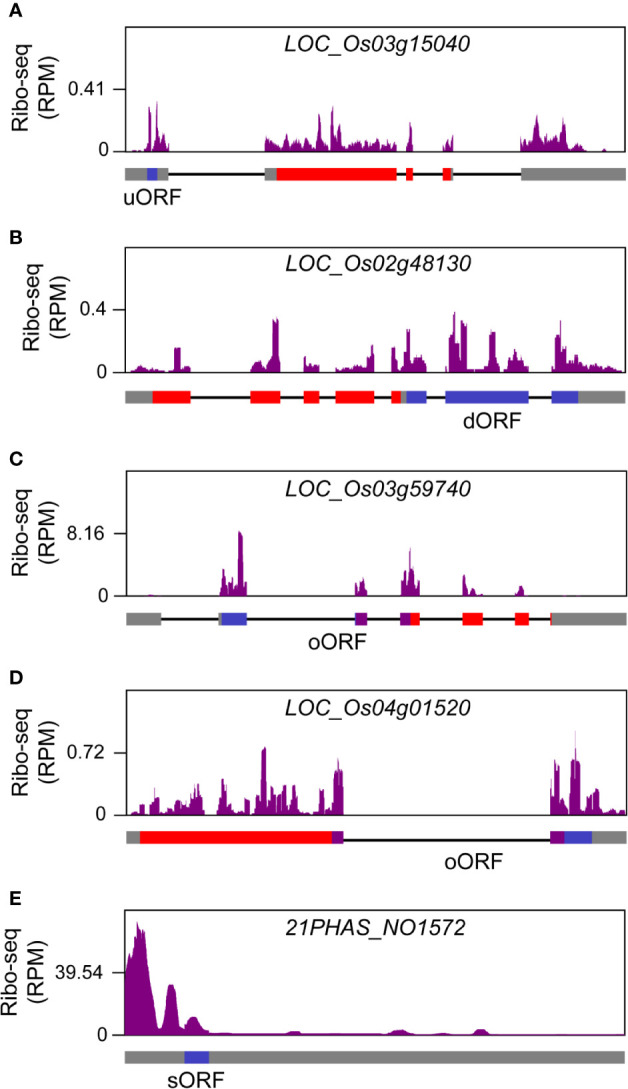
Novel open reading frames (ORFs) identified in rice genome. **(A)** A representative upstream ORF (uORF) that was located at the upstream region of annotated gene *LOC_Os03g15040*. **(B)** A representative downstream ORF (dORF) that was located at the downstream region of annotated gene *LOC_Os02g48130*. **(C)** A representative overlapped ORF (oORF) that was overlapped with the upstream region of annotated gene *LOC_Os03g59740*. **(D)** A representative oORF that was overlapped with the downstream region of annotated gene *LOC_Os04g01520*. **(E)** A representative small ORF (sORF) from *21PHAS_NO1572*, a previously reported long non-coding RNA that is located from 11,413,259 to 11,413,848 in rice chromosome 12 and can yield 21-nt phasiRNAs (Fei et al., 2016). The filled rectangles in gray, red, blue and purple, and the black lines in gene models that are provided at the bottom of ribo-seq panels represent untranslated regions (UTRs), exons, novel ORFs, overlapping regions between novel ORFs and annotated genes, and introns, respectively.

Small ORFs (sORFs) are ORFs equal to or smaller than 300 nt (100 amino acids) and have been ignored for a long term because of limitations in annotation methodology (Chugunova et al., 2018). Recently ribo-seq provided evidences for genome-wide existence of sORFs in fungi (Erpf and Fraser, 2018), animals (Mackowiak et al., 2015) and plants (Hellens et al., 2016). Based on their distribution in the genome, sORFs can be categorized into four major groups: uORFs that are located upstream of the annotated mORFs, downstream ORFs (dORFs) that are located downstream of annotated mORFs, overlapped ORFs (oORFs) that are overlapped with annotated mORFs partially or completely, and sORFs in canonical non-coding RNAs (Couso and Patraquim, 2017). We pooled the footprints from all libraries to predict potential sORFs. Novel ORFs, which belonged to uORF, dORF, oORF and non-coding RNA-derived ORF groups respectively, were identified in rice. The representative examples for each type of ORFs were shown in **Figure 9**. Identification of these novel ORFs will help to improve rice annotation, and studies on the functions and regulatory mechanisms of these ORFs will facilitate our understanding of translation control in rice.

### Troubleshooting

▪All solutions, reagents, pipette tips and tubes that are used in this protocol must be DNase/RNase-free
▪Polysome extraction buffer should be used immediately after being prepared and the concentration of each component should be strictly quantified, particularly for deoxycholic acid, of which high concentration could result in precipitation of ions in the buffer and failure of ribosome footprint isolation.
▪For storage, the polysome extracts are better to be kept at −80 – −65°C immediately after being frozen with liquid nitrogen.
▪It is suggested to use fresh polysome extracts for polysome profile analysis.
▪The amount of nuclease and digestion time could be influenced by tissue types and thus optimizations should be done if necessary.
▪Avoid excess UV light exposure when visualizing the SYBR Gold-stained PAGE gel.
▪Positive control should be included when PAGE purification is done for rice ribosome footprints or library construction to facilitate the recovery of samples with expected size.
▪For the enrichment of library by PCR amplification, the amount of templates and cycle number should be optimized and excessive templates or PCR cycles have to be avoided.

## Data Availability Statement

The datasets presented in this study can be found in online repositories. The names of the repository/repositories and accession number(s) can be found below: https://www.ncbi.nlm.nih.gov/, PRJNA523300.

## Author Contributions

LL, XY, YY, and BM designed research. XY and JC performed experiments. XY, BS, and LL analyzed data. XY and LL wrote the manuscript. All authors contributed to the article and approved the submitted version.

## Funding

This work was supported by Guangdong Innovation Research Team Fund (2014ZT05S078), Shenzhen Grant Plan for Science and Technology (JCYJ20190808112207542), Shenzhen High-level Talents Research Fund (827/000256), Natural Science Foundation of Guangdong Province (2018A030310446) and China Postdoctoral Science Foundation (2017M612741).

## Conflict of Interest

The authors declare that the research was conducted in the absence of any commercial or financial relationships that could be construed as a potential conflict of interest.

## Supplementary Material

Supplementary Table S1 Complete lists of gene ontology (GO) terms for the genes that are expressed discordantly at transcription and translation levels in seedling shoots of “Nipponbare” (NB) under salt stress.

Supplementary Figure S1 Size selection of PAGE-purified rice ribosome footprints. The red open rectangle indicates the recovered gel slices that contain around 28 nt – 30 nt rice ribosome footprints from seedling shoots of “Nipponbare” (NB) and “Sea Rice 86” (SR86) under normal growth condition (0 h) and after 24-h salt stress treatment (24 h).

Supplementary Figure S2 Recovery of PAGE-purified rice ribosome footprint libraries. The red open rectangle indicates the recovered gel slices that contain 70 nt – 90 nt rice ribosome footprint libraries constructed with seedling shoots of “Nipponbare” (NB) and “Sea Rice 86” (SR86) under normal growth condition (0 h) and after 24-h salt stress treatment (24 h). The bands at the bottom of the gel are adapter-dimers.

Supplementary Figure S3 PAGE purification and recovery of the enriched rice ribosome footprint libraries. The target bands ranging between 140 nt – 160 nt are indicated by the red open rectangle and cut out for recovery of the enriched rice ribosome footprint libraries constructed with seedling shoots of “Nipponbare” (NB) and “Sea Rice 86” (SR86) under normal growth condition (0 h) and after 24-h salt stress treatment (24 h). The bands under the target products are primer-dimers.

Supplementary Figure S4 Accordant changes of gene expression at transcription and translation levels in seedling shoots of “Nipponbare” (NB) under salt stress. **(A)** Expression of a representative gene (*LOC_Os01g03390*) that was up-regulated at both transcription and translation levels in seedling shoots of NB after 24-h salt stress treatment (24 h) in comparison to that under normal growth condition (0 h). **(B)** RNA-seq and ribo-seq coverage for *LOC_Os01g03390* in seedling shoots of NB under normal growth condition (0 h) and after 24-h salt stress treatment (24 h). **(C)** Expression of a representative gene (*LOC_Os01g01120*) that was down-regulated at both transcription and translation levels in seedling shoots of NB after 24-h salt stress treatment (24 h) in comparison to that under normal growth condition (0 h). **(D)** RNA-seq and ribo-seq coverage for *LOC_Os01g01120* in seedling shoots of NB under normal growth condition (0 h) and after 24-h salt stress treatment (24 h). The gene expression displays as mean ± standard deviations of three biological repeats and the cutoff values for up-regulated or down-regulated genes are fold change >= 1.5 and *P*-value <= 0.01 (A and C). The filled rectangles in gray and red, and the black lines in gene models that are provided at the bottom of ribo-seq panels represent untranslated regions (UTRs), exons and introns, respectively (B and D). “RPM” is short for “reads per million”.

Supplementary Figure S5 Expression comparison of discordantly expressed genes at transcription and translation levels in seedling shoots of “Nipponbare” (NB) under salt stress. **(A)** Expression of a representative gene (*LOC_Os05g41800*) that was transcriptionally down-regulated but translationally up-regulated (Group I) in seedling shoots of NB after 24-h salt stress treatment (24 h) in comparison to that under normal growth condition (0 h). **(B)** Expression of a representative gene (*LOC_Os01g05470*) that was only translationally up-regulated (Group II) in seedling shoots of NB after 24-h salt stress treatment (24 h) in comparison to that under normal growth condition (0 h). **(C)** Expression of a representative gene (*LOC_Os01g01170*) that was only transcriptionally down-regulated (Group III) in seedling shoots of NB after 24-h salt stress treatment (24 h) in comparison to that under normal growth condition (0 h). **(D)** Expression of a representative gene (*LOC_Os01g01620*) that was only transcriptionally up-regulated (Group IV) in seedling shoots of NB after 24-h salt stress treatment (24 h) in comparison to that under normal growth condition (0 h). **(E)** Expression of a representative gene (*LOC_Os01g03730*) that was only translationally down-regulated (Group V) in seedling shoots of NB after 24-h salt stress treatment (24 h) in comparison to that under normal growth condition (0 h). **(F)** Expression of a representative gene (*LOC_Os10g35050*) that was transcriptionally up-regulated but translationally down-regulated (Group VI) in seedling shoots of NB after 24-h salt stress treatment (24 h) in comparison to that under normal growth condition (0 h). The gene expression displays as mean ± standard deviations of three biological repeats and the cutoff values for up-regulated or down-regulated genes are fold change >= 1.5 and *P*-value <= 0.01. “RPM” is short for “reads per million”.

Supplementary Figure S6 Accordant changes of gene expression at translation and protein levels in seedling shoots of “Nipponbare” (NB) under salt stress. **(A–C)** Expression of three representative genes (*LOC_Os01g42860* in A, *LOC_Os02g15860* in B and *LOC_Os02g44870* in C) that were up-regulated at both translation and protein levels in seedling shoots of NB after 24-h salt stress treatment (24 h) in comparison to that under normal growth condition (0 h). **(D–F)** Expression of three representative genes (*LOC_Os02g57290* in D, *LOC_04g58200* in E and *LOC_12g08760* in F) that were down-regulated at both translation and protein levels in seedling shoots of NB after 24-h salt stress treatment (24 h) in comparison to that under normal growth condition (0 h). The gene expression displays as mean ± standard deviations of three biological repeats and the cutoff values for up-regulated or down-regulated genes are fold change >= 1.5 and *P*-value <= 0.01. “RPM” is short for “reads per million”.
