# Supplementary figures and images for: Construction of High-Quality Rice Ribosome Footprint Library
Source: Front Plant Sci. 2020 Sep 4;11:572237. doi: 10.3389/fpls.2020.572237 (PMC7500414; doi:10.3389/fpls.2020.572237)

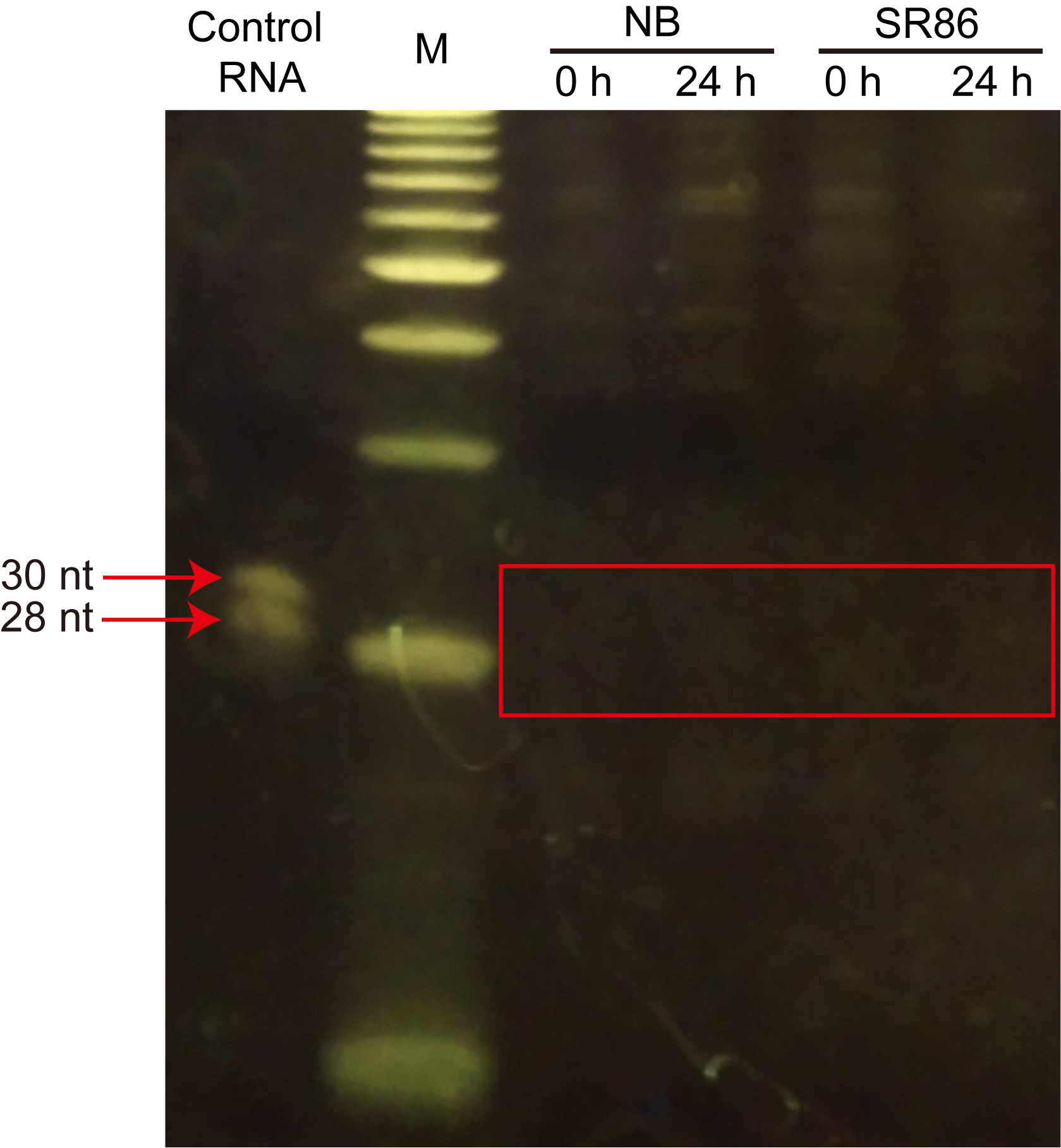

Supplement: Supplementary Figure S1 — Size selection of PAGE-purified rice ribosome footprints. The red open rectangle indicates the recovered gel slices that contain around 28 nt – 30 nt rice ribosome footprints from seedling shoots of “Nipponbare” (NB) and “Sea Rice 86” (SR86) under normal growth condition (0 h) and after 24-h salt stress treatment (24 h). [file Image_1.tif]

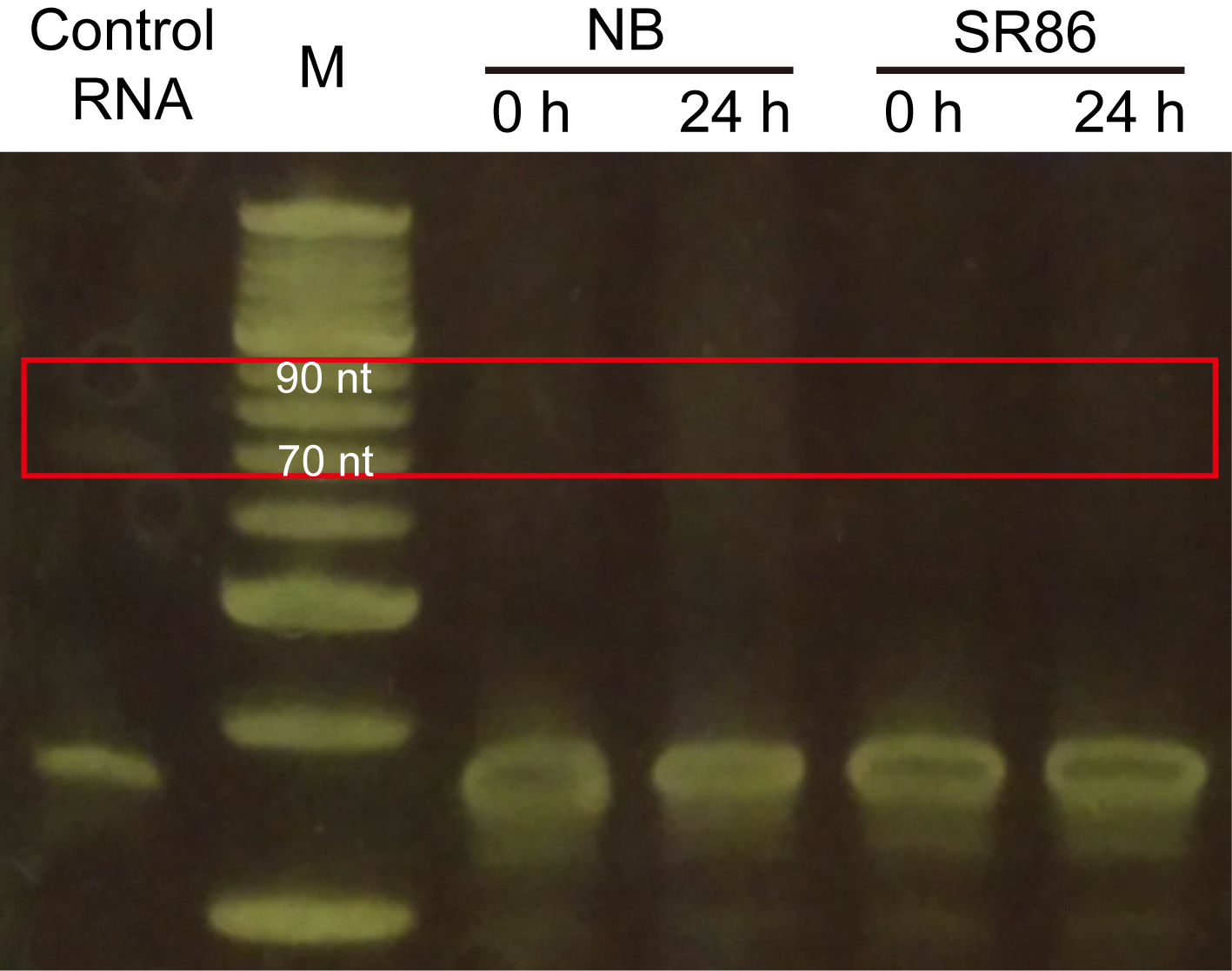

Supplement: Supplementary Figure S2 — Recovery of PAGE-purified rice ribosome footprint libraries. The red open rectangle indicates the recovered gel slices that contain 70 nt – 90 nt rice ribosome footprint libraries constructed with seedling shoots of “Nipponbare” (NB) and “Sea Rice 86” (SR86) under normal growth condition (0 h) and after 24-h salt stress treatment (24 h). The bands at the bottom of the gel are adapter-dimers. [file Image_2.tif]

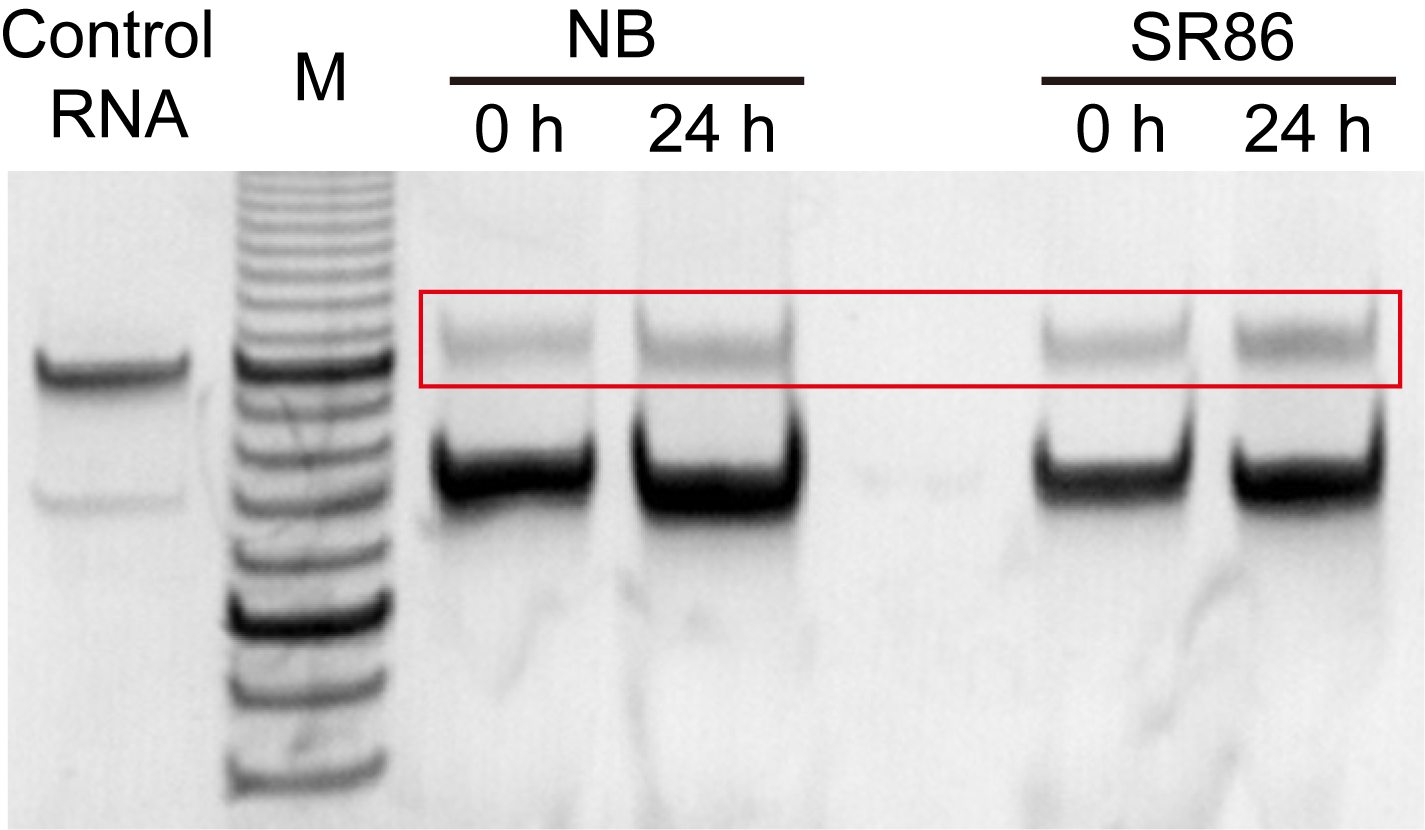

Supplement: Supplementary Figure S3 — PAGE purification and recovery of the enriched rice ribosome footprint libraries. The target bands ranging between 140 nt – 160 nt are indicated by the red open rectangle and cut out for recovery of the enriched rice ribosome footprint libraries constructed with seedling shoots of “Nipponbare” (NB) and “Sea Rice 86” (SR86) under normal growth condition (0 h) and after 24-h salt stress treatment (24 h). The bands under the target products are primer-dimers. [file Image_3.tif]

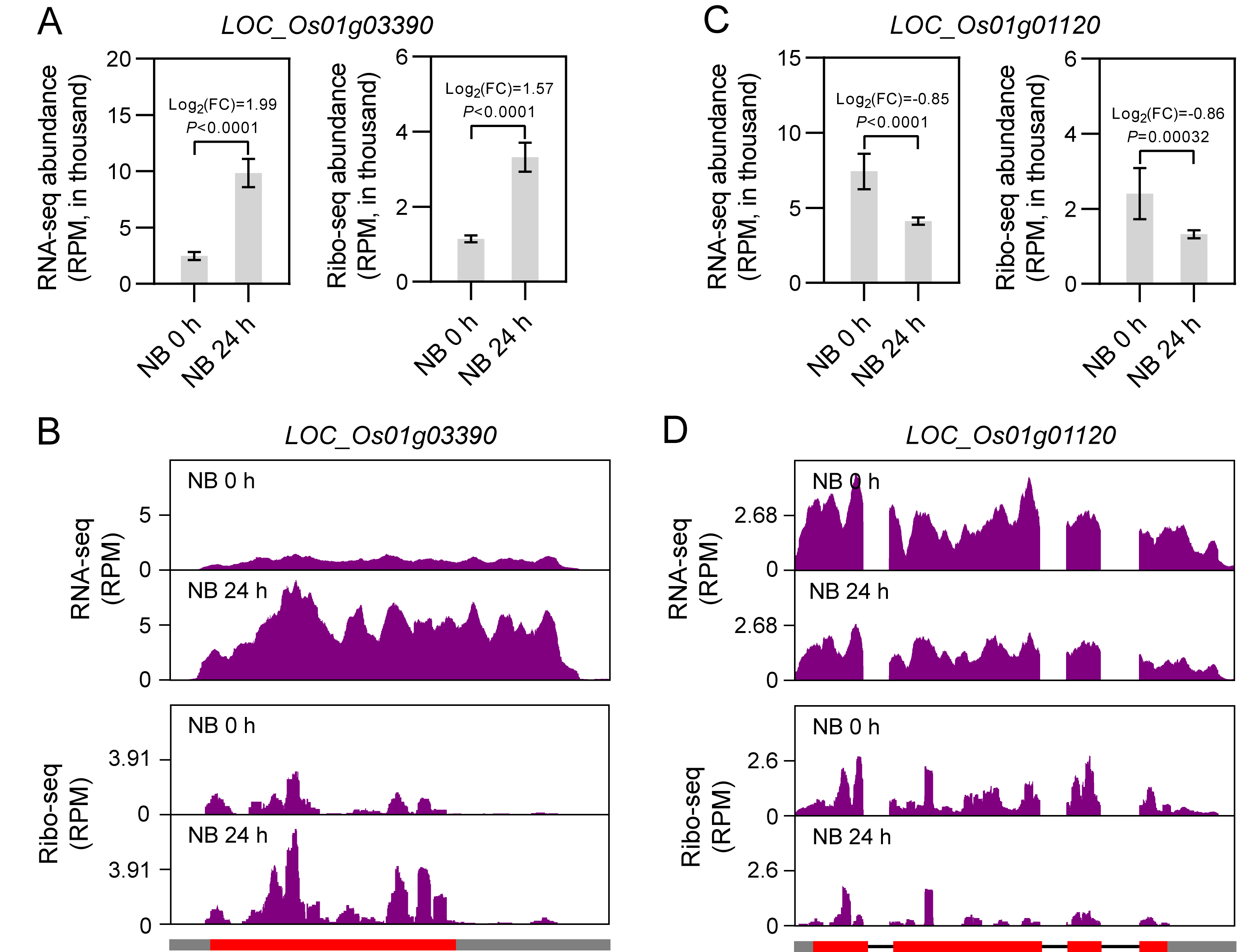

Supplement: Supplementary Figure S4 — Accordant changes of gene expression at transcription and translation levels in seedling shoots of “Nipponbare” (NB) under salt stress. (A) Expression of a representative gene (LOC_Os01g03390) that was up-regulated at both transcription and translation levels in seedling shoots of NB after 24-h salt stress treatment (24 h) in comparison to that under normal growth condition (0 h). (B) RNA-seq and ribo-seq coverage for LOC_Os01g03390 in seedling shoots of NB under normal growth condition (0 h) and after 24-h salt stress treatment (24 h). (C) Expression of a representative gene (LOC_Os01g01120) that was down-regulated at both transcription and translation levels in seedling shoots of NB after 24-h salt stress treatment (24 h) in comparison to that under normal growth condition (0 h). (D) RNA-seq and ribo-seq coverage for LOC_Os01g01120 in seedling shoots of NB under normal growth condition (0 h) and after 24-h salt stress treatment (24 h). The gene expression displays as mean ± standard deviations of three biological repeats and the cutoff values for up-regulated or down-regulated genes are fold change >= 1.5 and P-value <= 0.01 (A and C). The filled rectangles in gray and red, and the black lines in gene models that are provided at the bottom of ribo-seq panels represent untranslated regions (UTRs), exons and introns, respectively (B and D). “RPM” is short for “reads per million”. [file Image_4.tif]

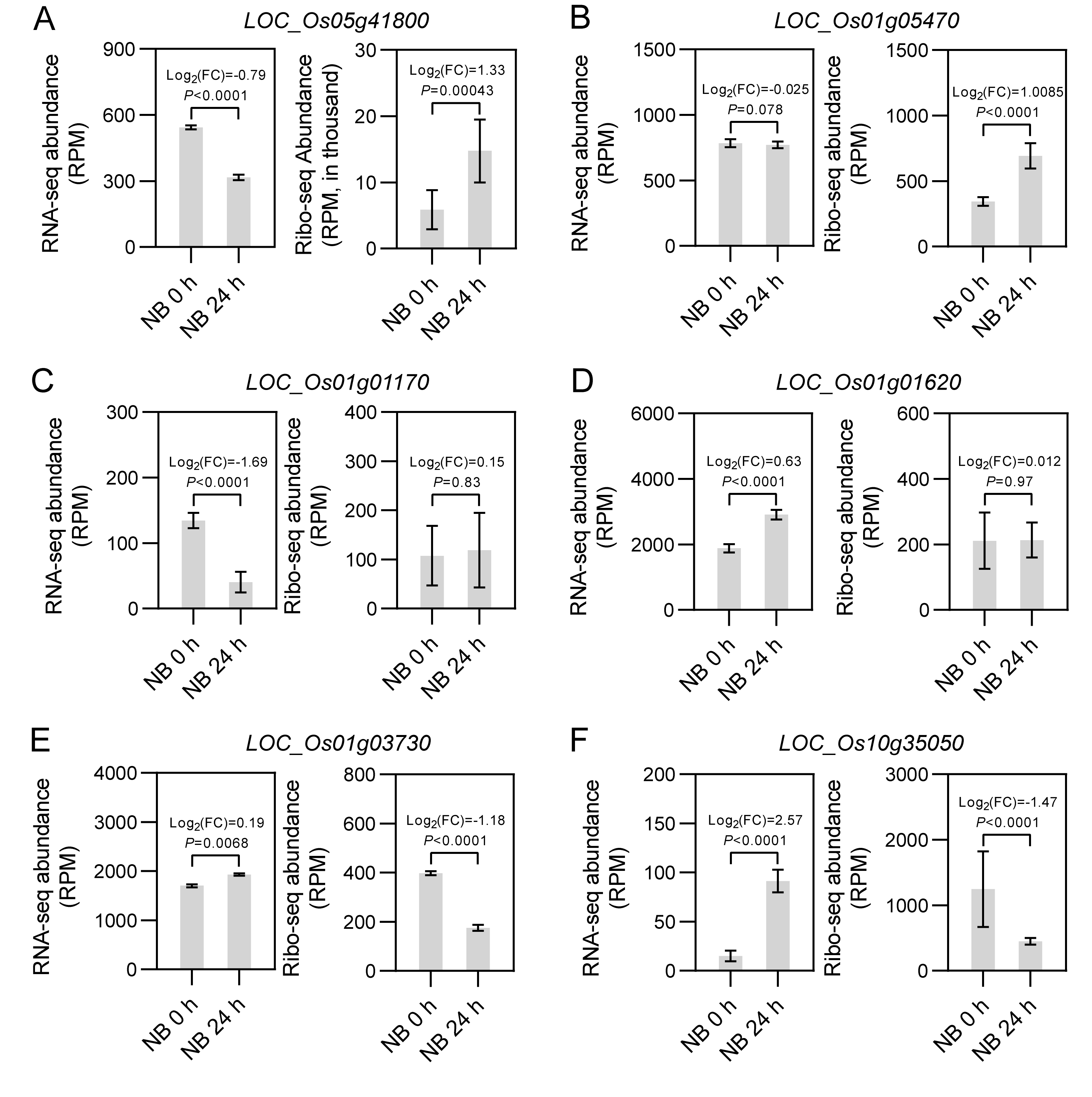

Supplement: Supplementary Figure S5 — Expression comparison of discordantly expressed genes at transcription and translation levels in seedling shoots of “Nipponbare” (NB) under salt stress. (A) Expression of a representative gene (LOC_Os05g41800) that was transcriptionally down-regulated but translationally up-regulated (Group I) in seedling shoots of NB after 24-h salt stress treatment (24 h) in comparison to that under normal growth condition (0 h). (B) Expression of a representative gene (LOC_Os01g05470) that was only translationally up-regulated (Group II) in seedling shoots of NB after 24-h salt stress treatment (24 h) in comparison to that under normal growth condition (0 h). (C) Expression of a representative gene (LOC_Os01g01170) that was only transcriptionally down-regulated (Group III) in seedling shoots of NB after 24-h salt stress treatment (24 h) in comparison to that under normal growth condition (0 h). (D) Expression of a representative gene (LOC_Os01g01620) that was only transcriptionally up-regulated (Group IV) in seedling shoots of NB after 24-h salt stress treatment (24 h) in comparison to that under normal growth condition (0 h). (E) Expression of a representative gene (LOC_Os01g03730) that was only translationally down-regulated (Group V) in seedling shoots of NB after 24-h salt stress treatment (24 h) in comparison to that under normal growth condition (0 h). (F) Expression of a representative gene (LOC_Os10g35050) that was transcriptionally up-regulated but translationally down-regulated (Group VI) in seedling shoots of NB after 24-h salt stress treatment (24 h) in comparison to that under normal growth condition (0 h). The gene expression displays as mean ± standard deviations of three biological repeats and the cutoff values for up-regulated or down-regulated genes are fold change >= 1.5 and P-value <= 0.01. “RPM” is short for “reads per million”. [file Image_5.tif]

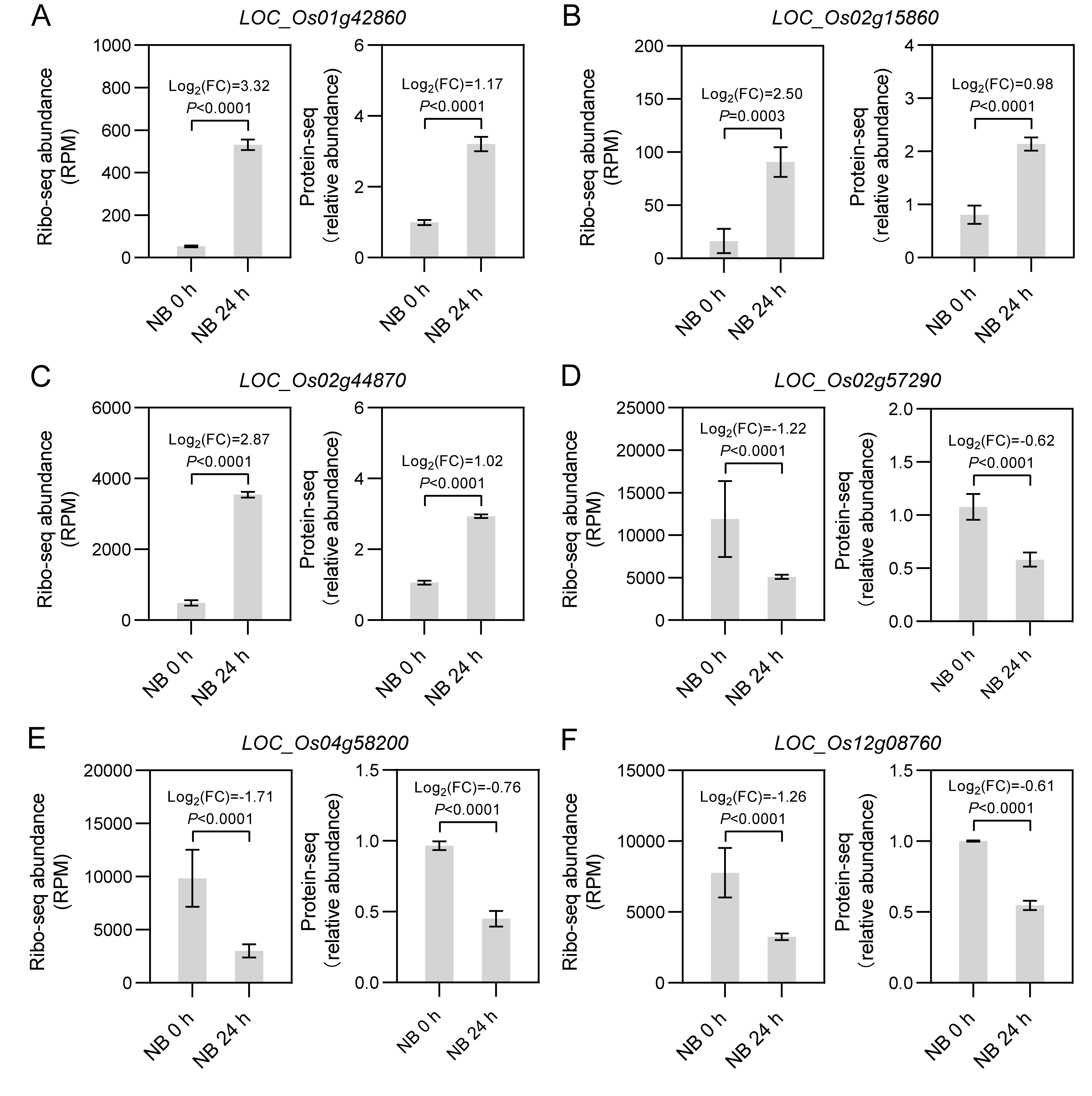

Supplement: Supplementary Figure S6 — Accordant changes of gene expression at translation and protein levels in seedling shoots of “Nipponbare” (NB) under salt stress. (A–C) Expression of three representative genes (LOC_Os01g42860 in A, LOC_Os02g15860 in B and LOC_Os02g44870 in C) that were up-regulated at both translation and protein levels in seedling shoots of NB after 24-h salt stress treatment (24 h) in comparison to that under normal growth condition (0 h). (D–F) Expression of three representative genes (LOC_Os02g57290 in D, LOC_04g58200 in E and LOC_12g08760 in F) that were down-regulated at both translation and protein levels in seedling shoots of NB after 24-h salt stress treatment (24 h) in comparison to that under normal growth condition (0 h). The gene expression displays as mean ± standard deviations of three biological repeats and the cutoff values for up-regulated or down-regulated genes are fold change >= 1.5 and P-value <= 0.01. “RPM” is short for “reads per million”. [file Image_6.tif]
